# A Medial Orbital Wall Fracture From Cricket Bat During Training: Surgical Approach

**DOI:** 10.7759/cureus.11021

**Published:** 2020-10-18

**Authors:** Ilinka Mis, Alexander Kapsokolis, Andromachi Fermanoglou, Maria Piagkou, Konstantinos Natsis

**Affiliations:** 1 Department of Ophthalmology, General Anticancer Oncological Hospital of Athens "Saint Savvas", Athens, GRC; 2 Anatomy and Surgical Anatomy, National and Kapodistrian University of Athens, Athens, GRC; 3 Anatomy and Surgical Anatomy, Aristotle University of Thessaloniki, Thessaloniki, GRC

**Keywords:** orbit, fracture, medial wall, training, athlete, cricket

## Abstract

A 24-year-old male athlete, injured by a cricket bat during training, was transferred to the hospital with an open head bleeding trauma in the medial part of his right eye. He was conscious, with no memory loss and dizziness. A medial orbital wall fracture (MOWF) with a foreign body presence was depicted in X-ray. Head computed tomography scan confirmed initial diagnosis and revealed the complete fracture of ethmoid sinuses. Temporary sutures were placed initially to close the wound and prevent trauma's infection. The ophthalmological exam revealed strabismus of the right eye due to medial rectus muscle entrapment and consequent diplopia in the horizontal gaze. After two weeks, surgery was planned to remove the foreign body, restore eye mobility, and correct diplopia. The wooden foreign body (2.5cm x 1cm) was removed with immediate decompression of the medial rectus muscle. The fracture was repaired by an open approach, a mesh net was placed and sutured to the periosteum for bone stabilization and regeneration. Fifteen days postoperatively, visual acuity was 10/10, ocular mobility was normal, and diplopia disappeared. Imaging is essential in diagnosis' confirmation and reconstructive surgery planning, without postoperative complications and complete patient's rehabilitation. The current report highlights the value of reconstructive surgery in orbital structures' preservation in complex MOWF cases. In such cases, the foreign body that entraps medial rectus muscle should be removed in time in order to prevent the orbital content displacement towards the gap created by the fractured bone with consequences enophthalmos and diplopia, as well as consecutive intraorbital infections. Eye preservation in the correct position eliminates strabismus.

## Introduction

The orbital wall fractures usually occur in the inferior or the medial wall. They result from traffic accidents, sports activities, violence, or fall injuries and may occur isolated or as part of a complex fracture. They are commonly associated with fractures of the orbital floor, as well as with fractures of the frontal, nasoethmoidal, and maxillary bone. The medial orbital wall, the thinnest part of the orbit, is rendered more susceptible to fracture. Surgical reconstruction of the medial orbital wall fractures (MOWF) is challenging because of the narrow operative field with complicated anatomical structures. In a limited space, many structures conjoin to form the medial orbital wall: the ethmoidal lamina papyracea, the lacrimal, sphenoid, and frontal bones, along with the anterior and posterior ethmoidal arteries and the optic nerve. The key point in such challenging surgeries is the pre-injury volume and orbital shape restoration with decompression of entrapped orbital muscular tissues to avoid post-traumatic enophthalmos, eye motility restriction, and consequent diplopia [1, 2]. Various reconstructive techniques of the medial orbital wall have been introduced to improve surgical outcomes and reduce complications.

Papers on MOWF management are scarce, revealing the difficulties of treating these injuries due to difficult manipulation of orbital contents and limitation of several types of materials in restoring the extremely complex three-dimensional (3D) anatomy of the medial orbital wall. Few studies highlighted the selected approach to treat MOWF by using prebent or porous polyethylene titanium meshes and implants with excellent outcomes in function and form [3-7].

Recent advances in endoscopic techniques allowed fractured medial orbital walls to be repositioned with excellent visualization and complete dissection of the circumference through the endoscopic transnasal approach [8].

The aim of reconstructive surgery is the preservation of the orbital structure to prevent the orbital content displacement towards the gap created by the fractured bone with probable consequences of enophthalmos and diplopia, and the removal of a foreign body that entrapped the medial rectus muscle to avoid consecutive intraorbital infections and to restore the eye to its correct position, eliminating the strabismus.

## Case presentation

**Figure 1:**
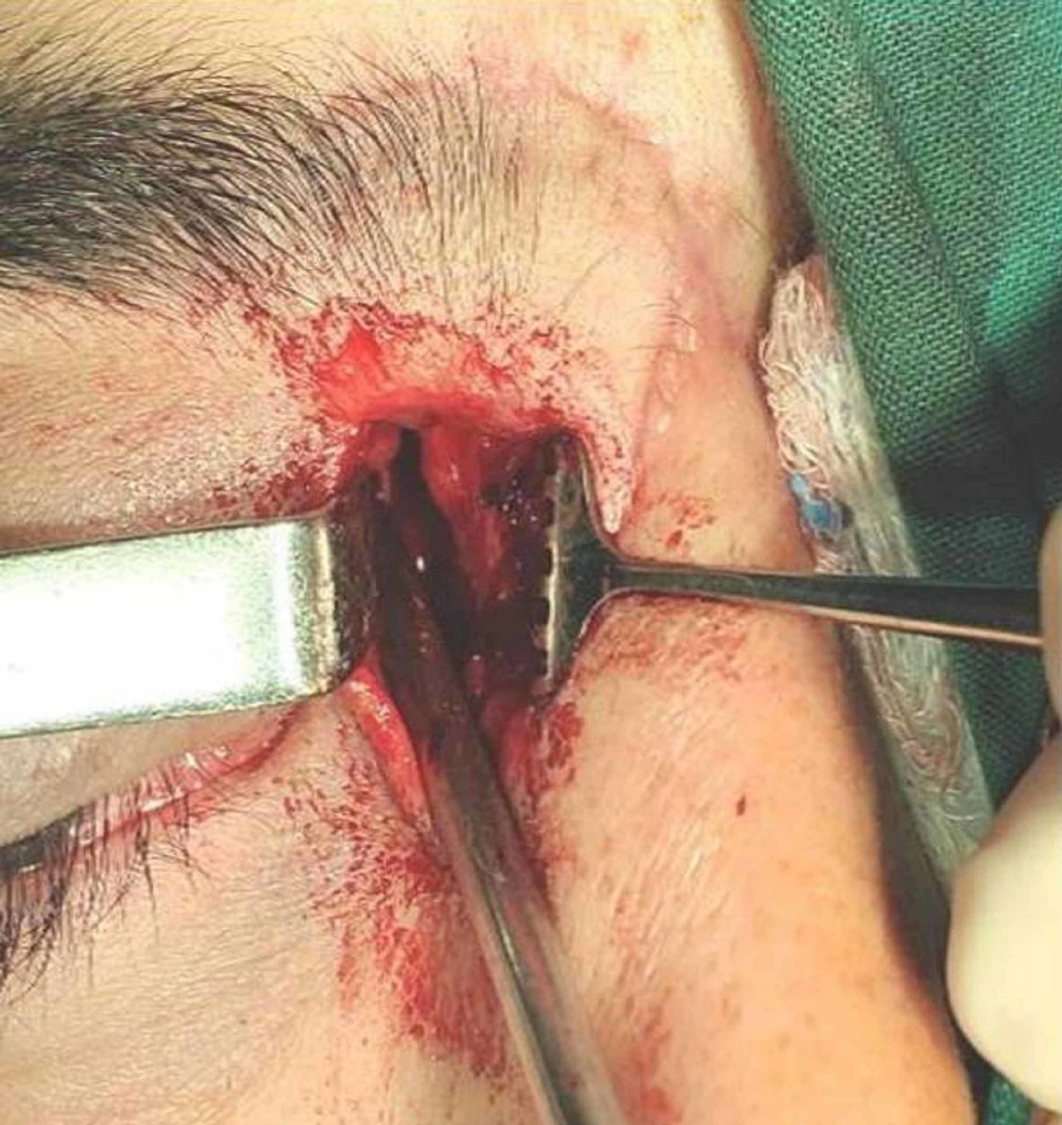
Skin incision at the medial canthal tendon level

A 24-year-old male athlete was injured by a cricket bat during training. Initially, he was transferred to the local hospital for an open bleeding trauma evaluation and management in the medial part of the right eye. The athlete was conscious, with no memory loss or dizziness. Neurological examination, blood pressure, and blood tests were normal. The X-ray revealed a MOWF and a foreign body presence in the right orbit. Temporary sutures were placed initially to close the wound and prevent trauma's infection. The patient was referred to our hospital for further evaluation and treatment. The preoperative ophthalmological examination confirmed swollen and bruised eyelids, conjunctiva chemosis, strabismus of the right eye due to medial rectus muscle entrapment, diplopia in the horizontal gaze, intraorbital pressure (IOP) 16/14 mmHg, and fundus without abnormalities. Imaging (head CT) scan revealed a MOWF and medial rectus entrapment by the presence of a foreign body in the right orbit. The coexistence of a complete fracture of the ethmoidal sinuses was found. The eyeball, sclera, and optic nerve were intact. The surgery was performed two weeks after the initial evaluation, via a curvilinear skin incision (20mm in length) at the medial canthal tendon level (Figure 1).

**Figure 2:**
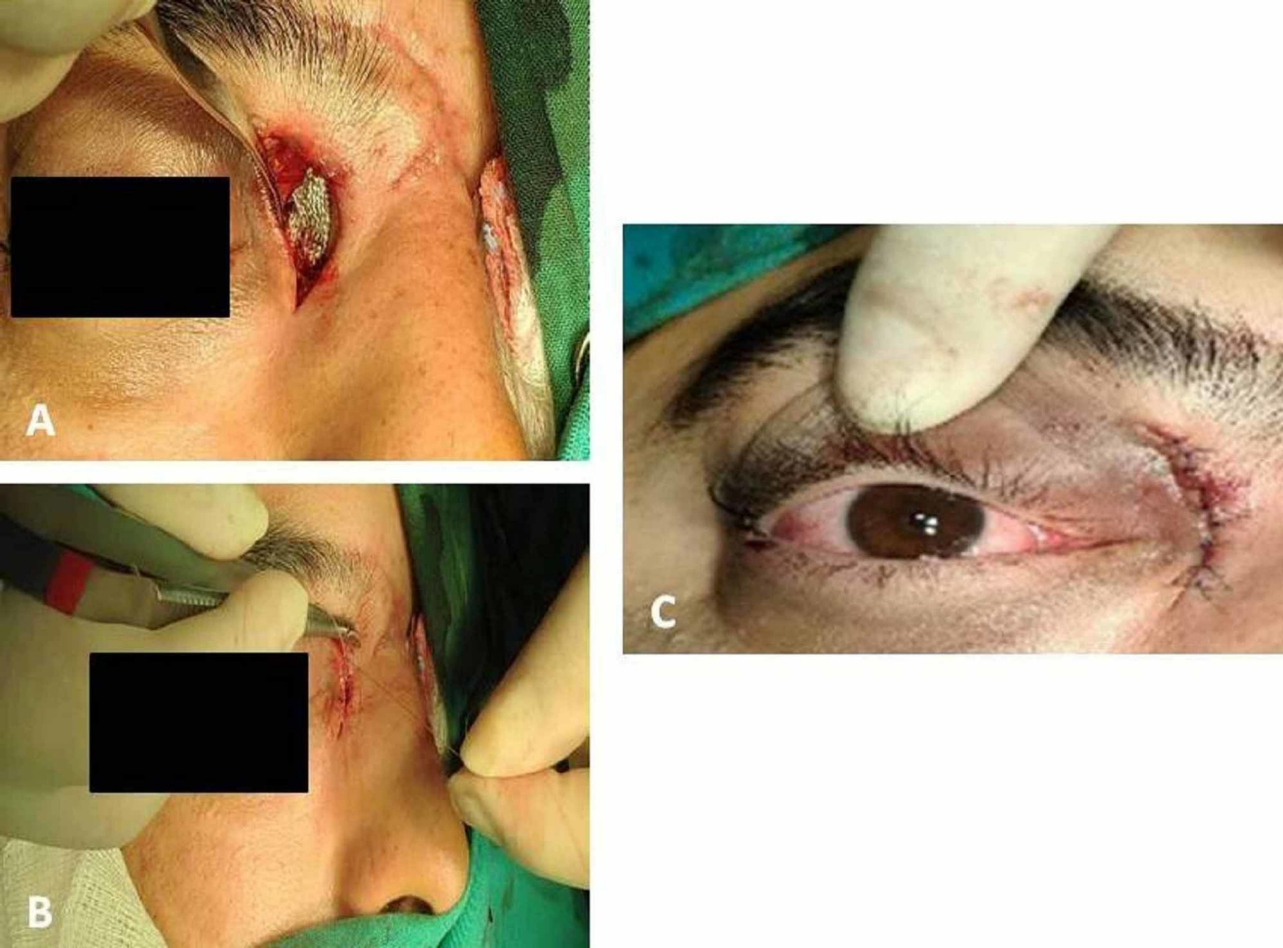
Mesh net placement and skin incision closure view A-B: a mesh net was placed and sutured with 5-0 vicryl to the periosteum for bone stabilization and regeneration. The skin incision was closed with a 6-0 prolene suture. C: view immediately after surgery.

The MOWF was revealed, and a small part of the destructed orbital bone was removed. A wooden foreign body (2.5cm x 1cm) was removed with immediate decompression of the medial rectus muscle and consecutive elimination of the strabismus. The fracture was repaired by an open approach, and a mesh net was placed and sutured to the periosteum for bone stabilization and regeneration (Figure 2).

**Figure 3:**
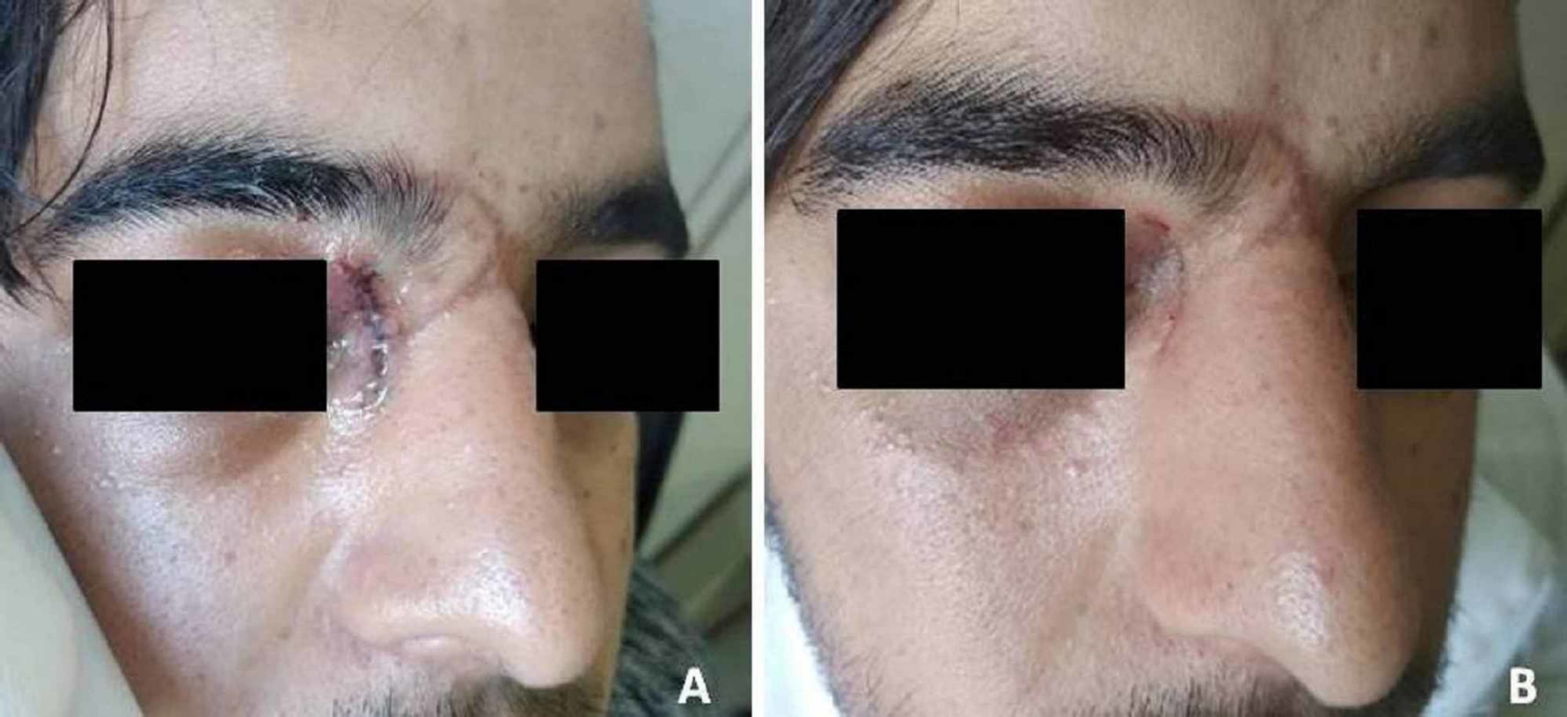
Patient's postoperative view The patient is on the first (A) and 15th (B) days postoperatively.

The patient returned 15 days after surgery for follow-up (Figure 3); his visual acuity was 10/10, ocular mobility was normal, and diplopia disappeared.

## Discussion

MOWF clinical manifestations are usually less severe than those of inferior wall fractures [9, 10]. However, they may cause complications, such as diplopia, enophthalmos, and extraocular muscles' entrapment [2, 11]. Enophthalmos may not appear immediately after trauma because soft tissue swelling may persist for weeks or months. The prevention of such complications is the accurate diagnosis and surgical reconstruction of the fractured wall. CT scan enables a more accurate diagnosis of the MOWF [2]. Surgical correction is generally necessary for patients with diplopia over a week, limited extraocular muscle movement, blurred vision from optic nerve compression, bone deficit greater than 2 cm, or enophthalmos resulting from orbital tissue herniation [11, 12].

The goal in reconstructive surgery of a MOWF is the recovery of undisturbed function and pre-injury globe position. Manson et al. pointed out the principles for a successful orbital reconstruction; they referred to the restoration of pre-injury anatomical shape and volume, with decompression and repositioning of prolapsed orbital soft tissue [7]. The complex soft tissue and bone anatomy of the medial orbit make much more challenging the exploration and identification of the posteromedial edge of the fracture, which is proximal to the orbital apex and relevant vital structures [12]. Visualization of the posteromedial orbit is very difficult with conventional transcutaneous access because medial canthal tendon insertion and the lacrimal system must be preserved [4]. Moreover, in medial orbital wall fractures, the surgeon had the additional technical difficulty of the anatomical reconstruction of the unique 3D anatomy with replication of slopes and curves, as well as difficulties in implant sizing and shaping [3].

Gerbino et al. [8], Bly et al. [13], and Markiewicz et al. [14] supported that navigation-aided surgery improves predictability and outcomes in complex orbital reconstruction. The use of titanium orbital mesh plates placed through a transconjunctival retrocaruncular approach allows safe and accurate orbital reconstruction in patients with MOWF. The use of endoscopic assistance through the surgical incisions improves the accuracy of treatment, allowing better visualization of the fractures' posterior edges.

## Conclusions

The current report highlights the value of reconstructive surgery in orbital structures' preservation in complex MOWF, as well as the removal of a foreign body that entraps medial rectus muscle should. Eye preservation in the correct position eliminates strabismus. Imaging and clinical experience are of paramount importance to confirm the diagnosis and plan reconstructive surgery without postoperative complications and complete patient rehabilitation.
